# Supplementary figures and images for: The noncoding small RNA SsrA is released by Vibrio fischeri and modulates critical host responses
Source: PLoS Biol. 2020 Nov 3;18(11):e3000934. doi: 10.1371/journal.pbio.3000934 (PMC7665748; doi:10.1371/journal.pbio.3000934)

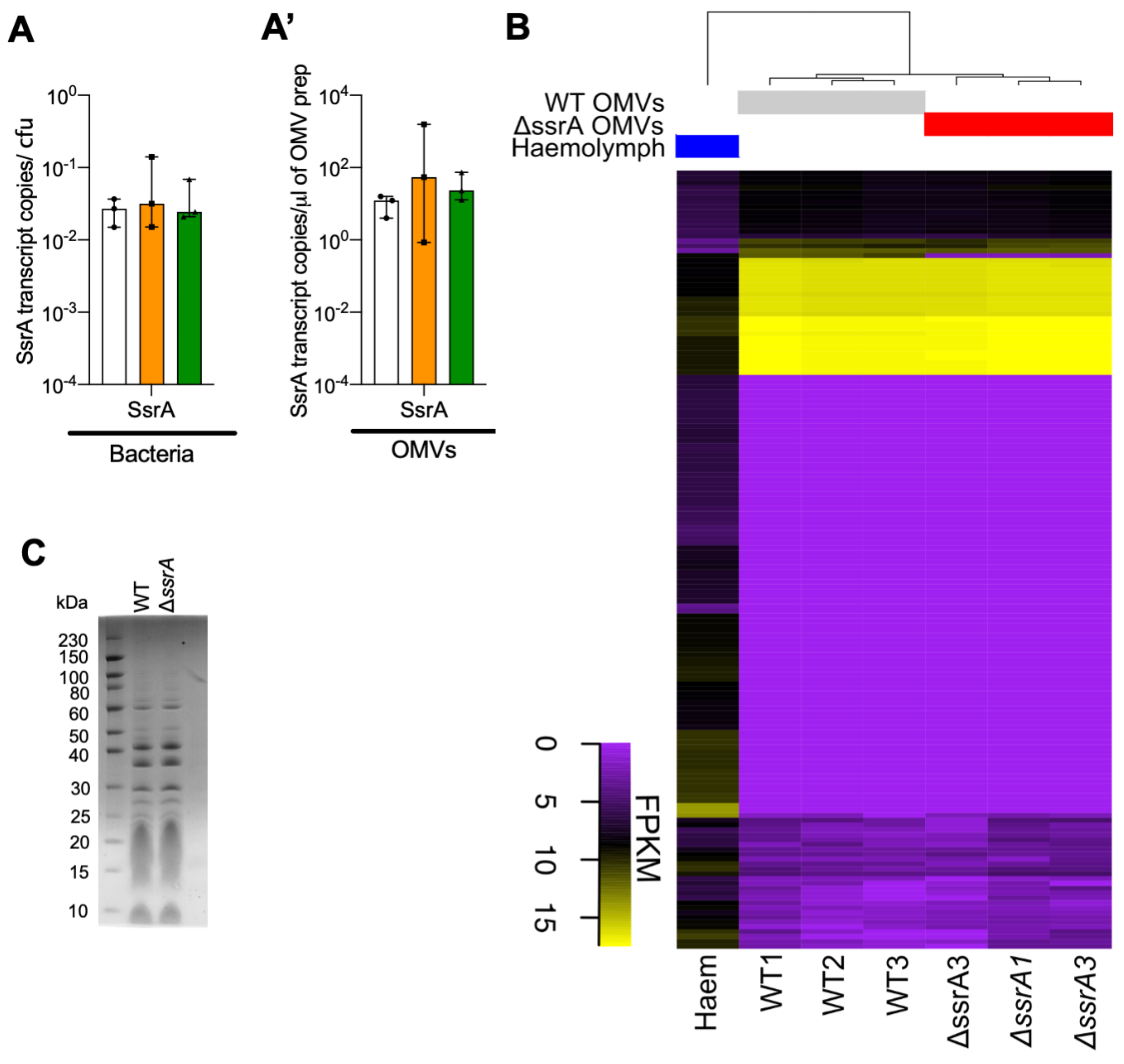

Supplement: S1 Fig — (A) qPCR measurements of SsrA expression by WT V. fischeri grown in three different media: a tryptone-based medium (LBS) or LBS with the addition of either glycerol (32.6 mM) or GlcNAc (10 mM). Data are presented as the number of transcript copies per cfu in late log phase (n = 3). (A’) qPCR measurements of SsrA within purified OMVs, presented as the number of transcript copies per volume of purified OMV preparation (n = 3). S6 Data. (B) Heat map of expression levels of V. fischeri RNA detected in squid hemolymph and in the RNA contents of OMVs. Hemolymph was collected from adult field-caught animals. OMVs were purified from cultures of WT V. fischeri or its ΔssrA derivative (S1 Data). (C) Soluble proteins present in purified OMVs isolated from cultures of WT or ΔssrA cells (S1 raw image). cfu, colony-forming units; GlcNAc, N-acetyl-glucosamine; LBS, Luria-Bertani salt medium; OMV, outer membrane vesicle; qPCR, quantitative PCR; sRNA, small RNA; WT, wild type. (TIFF) [file pbio.3000934.s001.tiff]

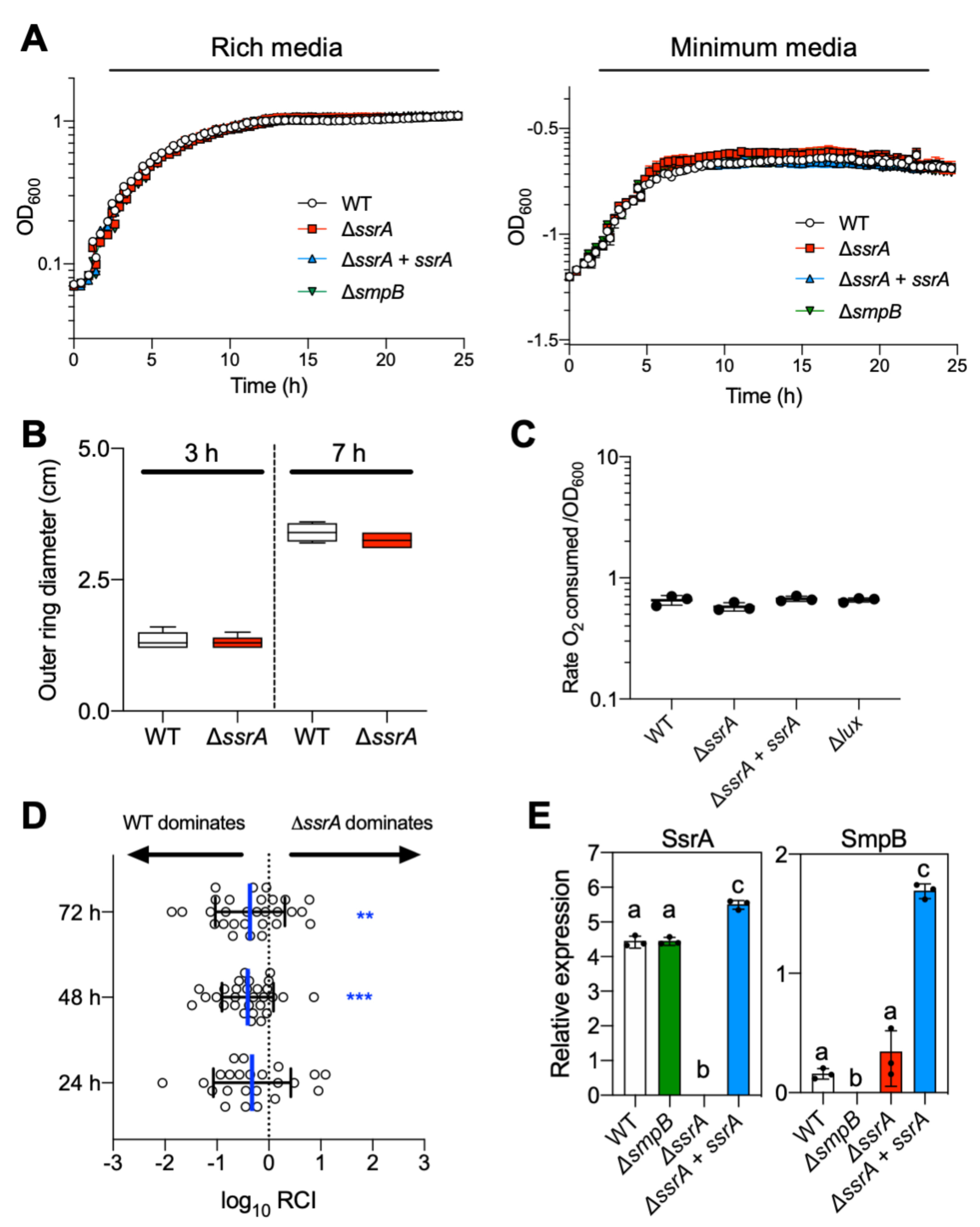

Supplement: S2 Fig — (A) Growth characteristics in (left) the tryptone-based medium LBS or (right) a minimal-salts medium, by the WT V. fischeri strain ES114 (WT), the ΔssrA mutant derivative, its genetic complement (ΔssrA + ssrA), and a deletion mutant (ΔsmpB) of the SsrA chaperone, SmpB. (B) Rates of motility in soft agar of WT or ΔssrA cells. The diameter of the outer ring was measured at 3 and 7 h post inoculation. Data are presented as the mean ± SD. (C) Normalized respiration rates of WT, ΔssrA, ΔssrA + ssrA, and a nonluminescent, lux-deletion mutant (Δlux) in SWT medium. Data are presented as the mean ± SD. (D) RCI between WT and ΔssrA in co-inoculated light organs after 24, 48, and 72 h. The RCI was calculated as the ratio of the two strains in the light organ, divided by their ratio in the inoculum. RCIs that are significantly different from zero are indicated (t test: **P value < 0.0071; ***P value < 0.0001). (E) Relative expression of ssrA and smpB transcripts by cells of WT and its mutant derivatives during the exponential phase of growth (OD600 between 0.65 and 0.74) in LBS medium. Expression was normalized to polymerase A and expressed as 2ΔΔCT. Significant differences are indicated by letters, based on a Bonferroni multiple-testing adjustment for pairwise comparisons. P value = 0.0083. The genetic complementation ΔssrA + ssrA, carries on a plasmid a copy of both ssrA and smpB. Numerical values found at S6 Data. LBS, Luria-Bertani salt medium; OD600, optical density at 600 nm; RCI, relative competitive index; WT, wild type. (TIFF) [file pbio.3000934.s002.tiff]

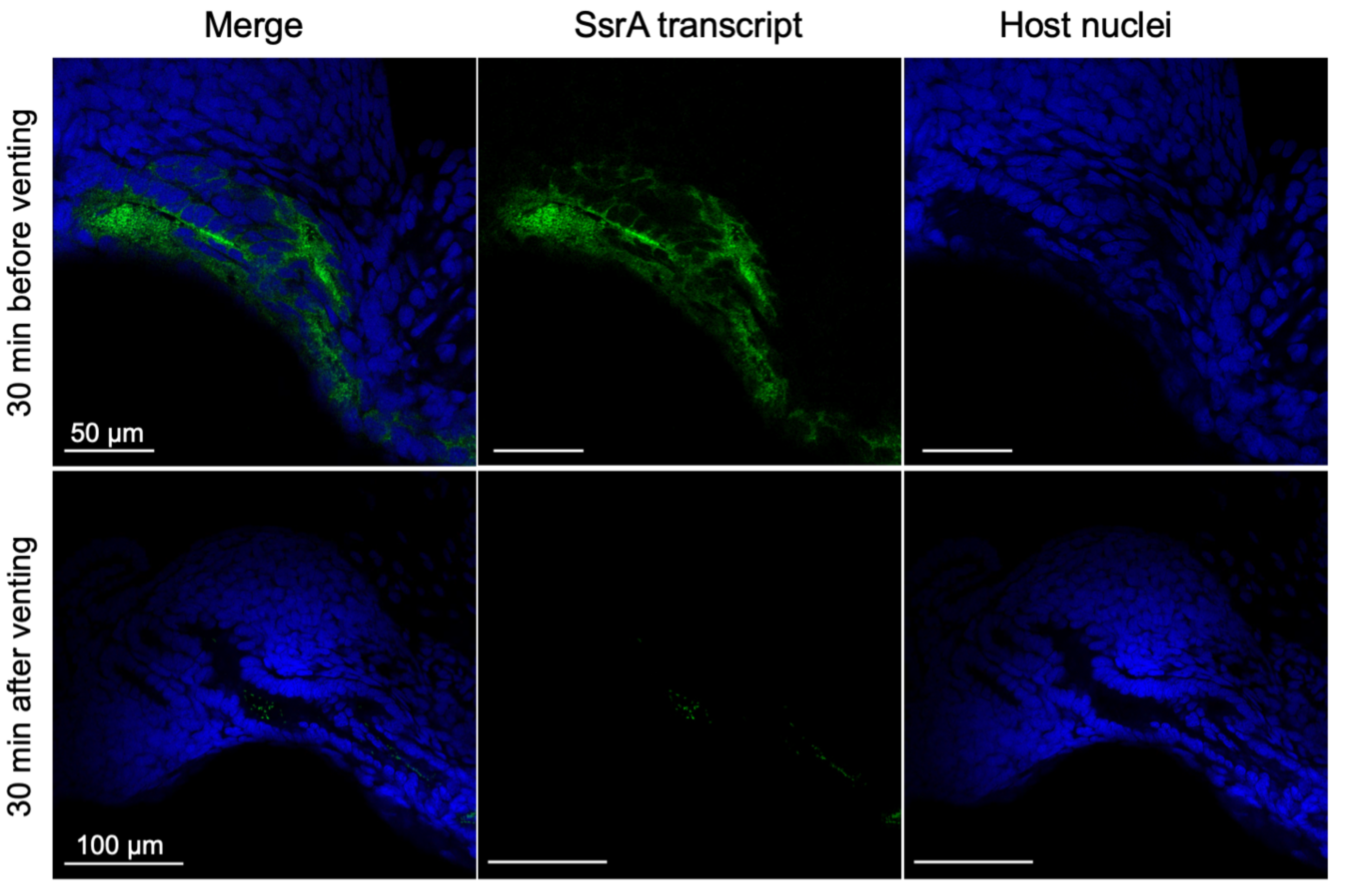

Supplement: S3 Fig — Representative confocal microscopy images localizing symbiont SsrA (green) by HCR 30 min before (top) or 30 min after (bottom) symbiont expulsion. Light organs were colonized by WT V. fischeri. HCR, hybridization chain reaction; WT, wild type. (TIFF) [file pbio.3000934.s003.tiff]

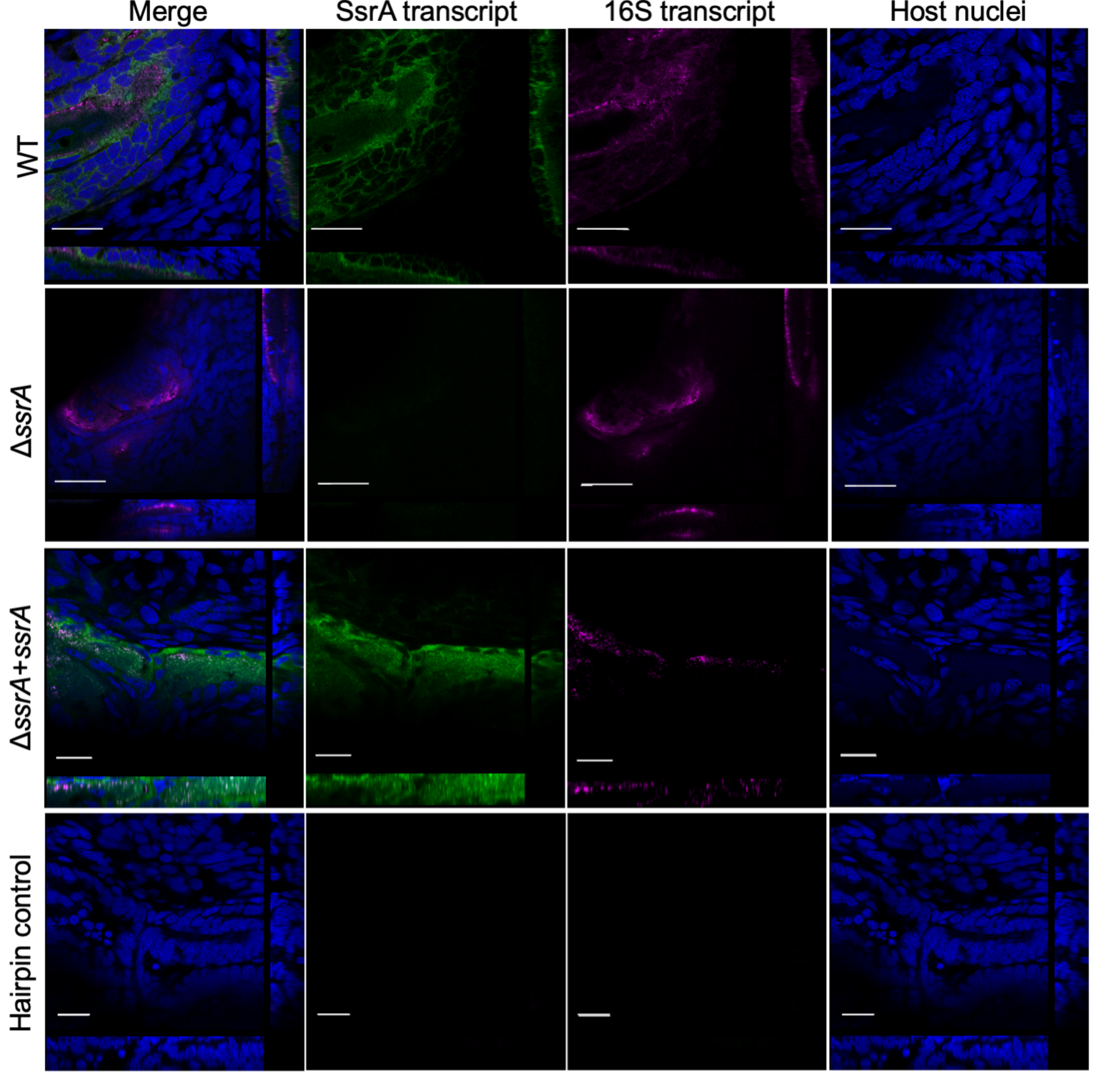

Supplement: S4 Fig — Representative confocal microscopy images with orthogonal projections localizing symbiont SsrA (green) and 16S (magenta) transcripts within the crypt epithelium of light organs colonized by WT, ΔssrA, or ΔssrA + ssrA, compared to the HCR hairpin negative control; host nuclei (blue). Scale bars = 20 μm. HCR, hybridization chain reaction; WT, wild type. (TIFF) [file pbio.3000934.s004.tiff]

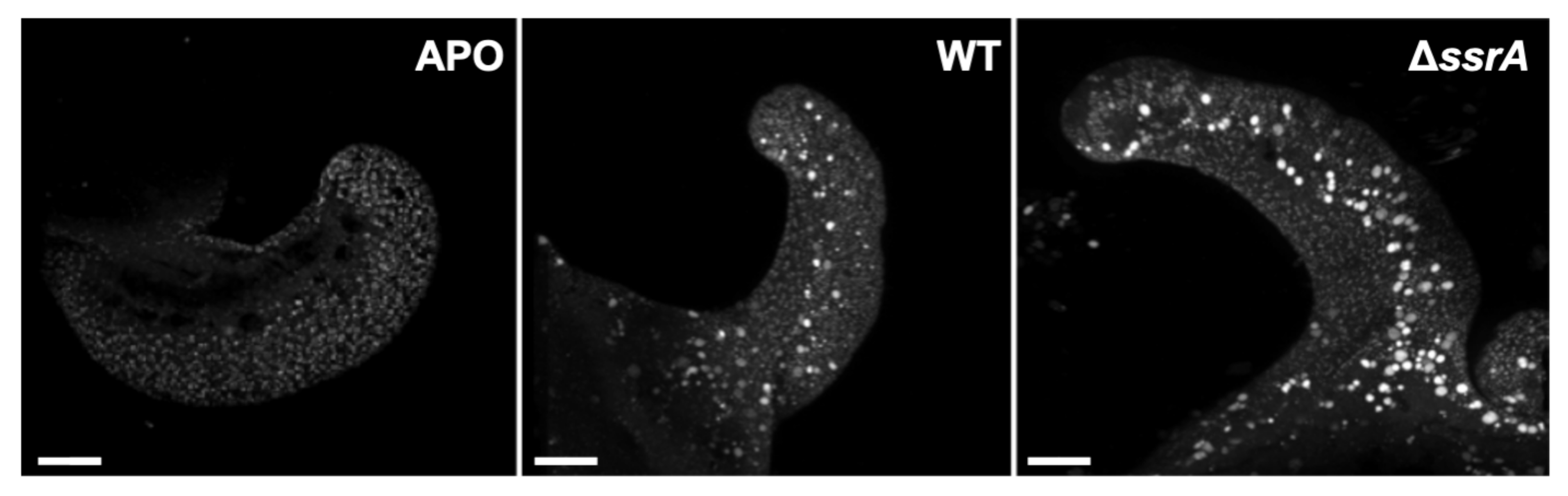

Supplement: S5 Fig — Representative confocal microscopy images of AO-stained juvenile light organs, after exposure to no (APO), WT, or ssrA-deletion mutant (ΔssrA) V. fischeri. Scale bar = 60 μm for all images. AO, acridine orange; APO, aposymbiotic; WT, wild type. (TIFF) [file pbio.3000934.s005.tiff]

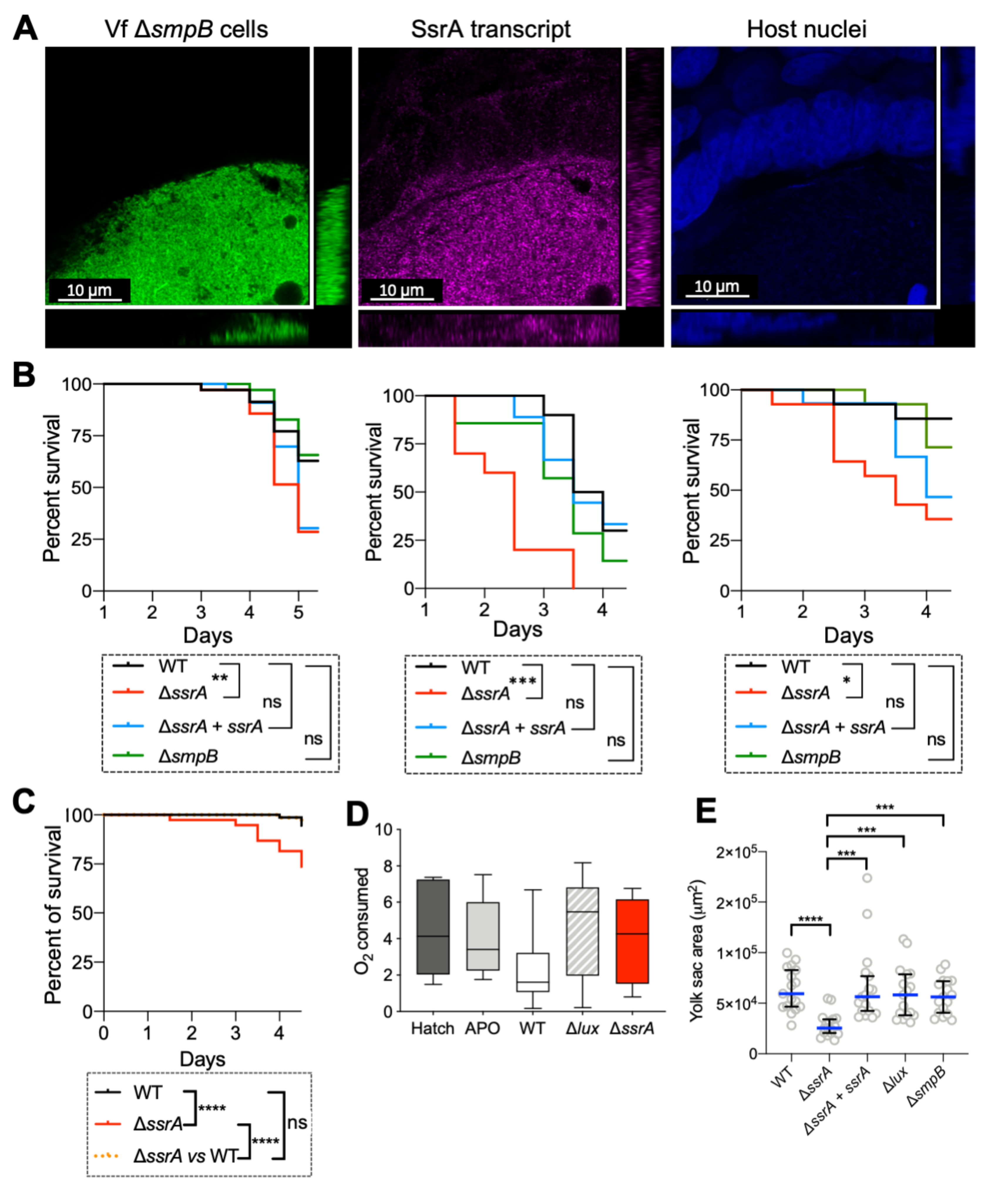

Supplement: S6 Fig — Visualization of SsrA transcript (magenta) in a whole-mount light organ, 24 h after colonization with a GFP-labeled ΔsmpB strain of V. fischeri (green). A representative confocal image indicates that symbiont SsrA transcript is within the crypt epithelial cells. Scale bar = 10 μm. (A) Visualization by HCR of SsrA transcript (magenta) in crypt #1 of a whole-mount light organ, 24 h after colonization with a GFP-labeled ΔsmpB strain of V. fischeri (green), including orthogonal views of a confocal microscopy Z-stack; host nuclei (TO-PRO-3, blue). (B) Kaplan-Meier survival plots of juvenile squid colonized by WT, ΔssrA, its genetic complement (ΔssrA + ssrA), or the ΔsmpB strain. Data are from replicate #1 (left), #2 (middle), or #3 (right). Survival-curve analyses used the log-rank Mantel-Cox test, with Bonferroni multiple-testing adjustment for pairwise comparisons. P value = 0.016 (S3 Data). (C) Kaplan-Meier survival plots of juvenile squid that were either single-colonized by WT or ΔssrA or co-colonized at a 1:1 inoculum ratio with both WT and ΔssrA (n = 60); note that the WT and co-colonized data are coincident and significantly different from ΔssrA. Log-rank Mantel-Cox test, with Bonferroni multiple-testing adjustment for pairwise comparisons. P value = 0.016 (S7 Data). (D) Respiration rates of newly hatched squid (“Hatch,” n = 5) or of animals after 24 h, that were either maintained APO (n = 12) or colonized by WT (n = 12), ΔssrA (n = 11), or Δlux (n = 11) strains. No significant difference between treatments was noted (S7 Data). (E) Internal yolk-sac areas, 2 d post colonization with WT, ΔssrA, its complement (ΔssrA + ssrA), the dark-mutant (Δlux) or ΔsmpB strains. Analysis used Kruskal–Wallis ANOVA, followed by Dunn’s multiple comparison test (n = 17). Data are represented as the median, with 95% confidence interval. P value code: ****<0.0001; ***<0.0002; **<0.001; *<0.021. ns: nonsignificant for all figures (S7 Data). APO, aposymbiotic; GFP, green fluorescent pr [file pbio.3000934.s006.tiff]

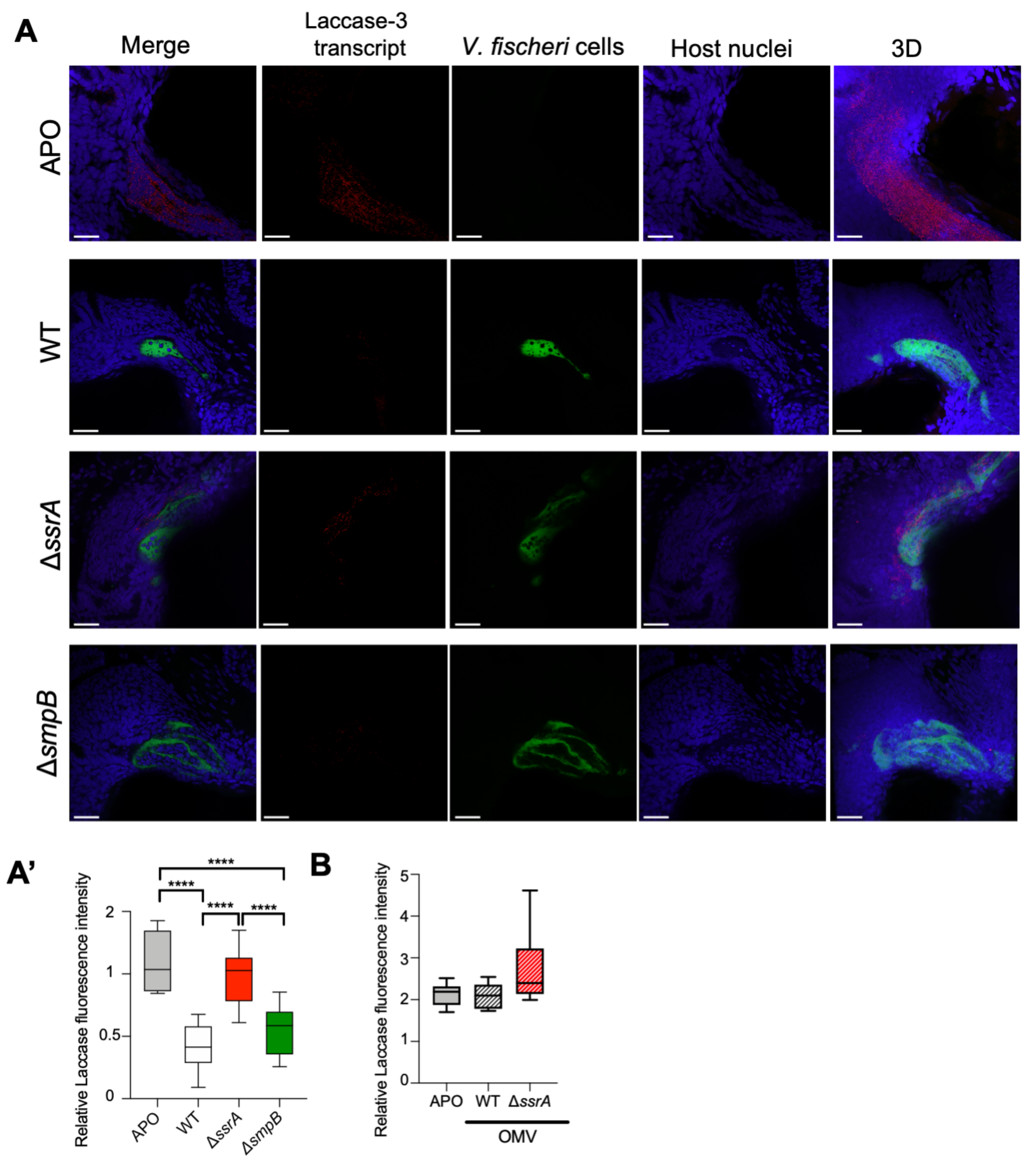

Supplement: S7 Fig — (A) Localization of the laccase-3 transcript (magenta) in whole-mount light organs, 24 h post colonization. Representative confocal images showing laccase-3 expression in the crypt epithelia of APO (uncolonized) and WT, ΔssrA or ΔsmpB-colonized light organs; merged mid-section of Z-stack, and 3D reconstruction of the stack (S7 Data). (A’) Quantification of laccase-3 signal using relative fluorescence intensity of a Z-series image of the light organ. P values were calculated using a 1-way ANOVA with TMC. (B) Quantification of laccase-3 presence by HCR fluorescence signal intensity from a Z-series of light organs (n = 5), 3 h after incubation with OMVs isolated from either WT or ΔssrA cultures. Addition of symbiont OMVs by themselves does not significantly change the expression of laccase-3 in the crypt epithelium (S7 Data). APO, aposymbiotic; HCR, hybridization chain reaction; TMC, Tukey’s multiple comparison test; WT, wild type. (TIFF) [file pbio.3000934.s007.tiff]

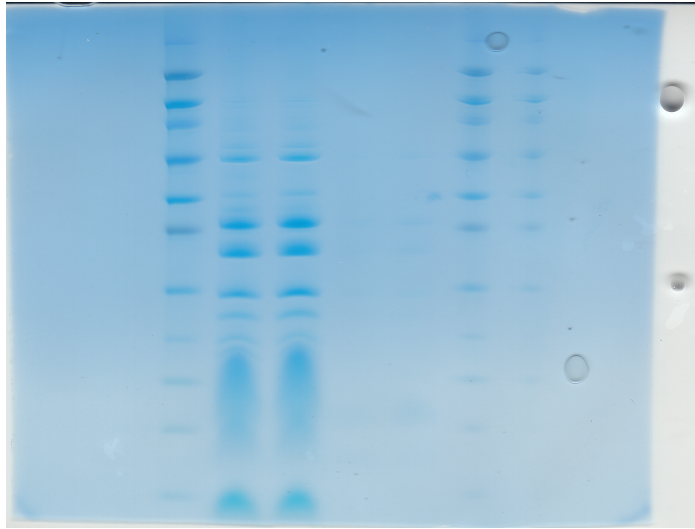

Supplement: S1 Raw image — OMV, outer membrane vesicle. (PDF) [file pbio.3000934.s009.pdf]
