# Supplementary material for: The noncoding small RNA SsrA is released by Vibrio fischeri and modulates critical host responses
Source: PLoS Biol. 2020 Nov 3;18(11):e3000934. doi: 10.1371/journal.pbio.3000934 (PMC7665748; doi:10.1371/journal.pbio.3000934)
Supplement: S1 Table — (DOCX) [file pbio.3000934.s008.docx]

Table S1: Oligonucleotides information.

| **Oligonucleotide ID** | **Description** | **Sequence 5' to 3'** | **Source** |
| --- | --- | --- | --- |
| **Cloning primers** | | | |
| EcoRI-ssrAups-F2 | Up-stream SsrA amplification (PCRa)/PCRab to insert into pKV363 | GCATGGAATTCGGTTATCTAGTCAAACCACTTCCCT | This study |
| ssrAdel-BamHI-R6 | Up-stream SsrA amplification (PCRa) | GTACGGATCCAGCCCCAAAGTTTTGTTACCAG | This study |
| BamHI-ssrAdel-F7 | Down-stream SsrA amplification (PCRb) | CGATCGGATCCGGGTTTTTCGTTTTAATGGCG | This study |
| SsrAdown-XhoI-R3 | Down-stream SsrA amplification (PCRb)/PCRab to insert into pKV363 | CGTACCTCGAGTCTCTTCGTGGCGCTCATTT | This study |
| KpnI-ssrAcomp-F1 | SsrA and smpB amplification to insert into pVSV105 | GCATGGGTACCGACTAAATCGTACATCTGCTTTG | This study |
| XbaI-ssrAcomp-R2 | SsrA and smpB amplification to insert into pVSV105 | CGTACTCTAGACCCCTGTAACCTATTGATTAC | This study |
| ApaI-smpB_A-F1 | Up-stream smpB amplification (PCRa)/PCRab to insert into pSMV3 | CGATCGGGCCCCGTTACGGCTAAACACACCG | This study |
| BamHI-smpB_A-R1 | Up-stream smpB amplification (PCRa) | CGATCGGATCCTGCCATAATGCCCACATTATAC | This study |
| BamHI-smpB_B-F1 | Down-stream smpB amplification (PCRb) | CGATCGGATCCAGTAGTTTGCGTTAATTTCAACCAG | This study |
| SpeI-smpB_B-R1 | Down-stream smpB amplification (PCRa)/PCRab to insert into pSMV3 | CGTACACTAGTGTTAGAGATGGATAGCGTGG | This study |
| ***V. fischeri* qRT-PCR primers** | | | |
| ssrA_qF2 | Target gene *ssrA* amplification | AGCTCTCCTGCCCTAGCTTC | This study |
| ssrA_qR2 | Target gene *ssrA* amplification | ATCCGTTCTAGCTCGTTTGG | This study |
| smpB_qF | Target gene *smpB* amplification | CACACCGCTACAAGCAGCAT | This study |
| smpB_qR | Target gene *smpB* amplification | CTTCACGGTTTACACGGCCA | This study |
| polA_qF2 | Reference gene *polA* amplification | AGAGCCAAACGTAGATGAGTTGA | This study |
| polA_qR2 | Reference gene *polA* amplification | CGATCTTACCGTCGCTTCCA | This study |
| ***E. scolopes* qRT-PCR primers** | | | |
| 40S-qF3 | Reference gene *S19* ribosomal protein amplification | AAGGCTTTGTCCACCTTCCT | (Moriano-Gutierrez et al., 2019) |
| 40S-qR3 | Reference gene *S19* ribosomal protein amplification | TAAATGCTCCAACACCAGCA | (Moriano-Gutierrez et al., 2019) |
| RIGI-3_F1q | Target gene *RIG-I* amplification | ACAGCAGCGTCCATCATCAA | This study |
| RIGI-3_R1q | Target gene *RIG-I* amplification | GGACCAGGTAAAGGACACGG | This study |
| laccase-3_F1q | Target gene *laccase* amplification | CTCCGTCCAATGAATGTGTG | This study |
| laccase-3_R1q | Target gene *laccase* amplification | TAGGGACAGAAAGCCGATGT | This study |
| C3_F1q | Target gene *complement 3* amplification | TGCTGTTCCGTTCTGTGAGCACTA | Collins et al 2012 |
| C3_R1q | Target gene *complement 3* amplification | GCAACACACTCTCTCTTTGAGCGCAT | Collins et al 2012 |
| CIKS_F1q | Target gene *CIKS* amplification | GGTGGAAGTGCCGATAACAT | This study |
| CIKS_R1q | Target gene *CIKS* amplification | TGCTGAAACCCATTTTAGGG | This study |
| **HCR probes version 2** | | | |
| *V. fischeri* 16S Probe #1 | Target 16S rRNA | TGTGCGGGCCCCCGTCAATTCATTTGAGTTTTAATCTTGCGACCGTACTC | (Nikolakakis et al 2015) |
| *V. fischeri* 16S Probe #2 | Target 16S rRNA | GTAGGTAAGGTTCTTCGCGTTGCATCGAATTAAACCACATGCTCCACCGC | (Nikolakakis et al 2015) |
| *V. fischeri* SsrA Probe #1 | Target SsrA RNA | ACGACATGCTCCTCGGGTTTCAAAATTCCCGTCGAATCCTGAATCAGCCC | This study |
| *V. fischeri* SsrA Probe #3 | Target SsrA RNA | CCGTCTTACAAGCAGAAGCTAGGGCAGGAGAGCTCTCAGCAGGTTATTAA | This study |
| *V. fischeri* SsrA Probe #4 | Target SsrA RNA | AATTCGAAGTTCATCTCTCAGGCGGGAGAATCCGTTCTAGCTCGTTTGGG | This study |
| *V. fischeri* SsrA Probe #5 | Target SsrA RNA | CGATCTTTGATTTCACCGTAAAACTGCGAACCGACACGCTATCTTATGGC | This study |
| **HCR probes version 3** | | | |
| *E. scolopes* Laccase-3 Probe #1 | Target Laccase-3 RNA | TGATGACGTCATCTATTGGAACCGGGTTCGCTGCACTCGTGTTAACGACTTC | This study |
| *E. scolopes* Laccase-3 Probe #2 | Target Laccase-3 RNA | GGATTGTTCCATTAACCGCCACAACAAGTCGTGACTCAGTGTAACCATCTAA | This study |
| *E. scolopes* Laccase-3 Probe #3 | Target Laccase-3 RNA | TCTCAACGTTGATAATTACAGTTTGGCCAACGTAGACTTCAATCGCCGGACC | This study |
| *E. scolopes* Laccase-3 Probe #4 | Target Laccase-3 RNA | CTTTCTGCTCCAATCCGTGCCAATGAATGGTAACGCCACTAGAGTAGAGATG | This study |
| *E. scolopes* Laccase-3 Probe #5 | Target Laccase-3 RNA | CAGGTCCTATAGGACACTGGGTTACATAGGGGACACCGTCCATAAACGGCGT | This study |
| *E. scolopes* Laccase-3 Probe #6 | Target Laccase-3 RNA | TGTAATCGCTGATGACCATAAGATGTTCGGGCATAGACATCGGCTTTCTGTC | This study |
| *E. scolopes* Laccase-3 Probe #7 | Target Laccase-3 RNA | CGTACATACCATGCACCATCTTAAGATAAGCTACATCAGATTCCCAATGATG | This study |
| *E. scolopes* Laccase-3 Probe #8 | Target Laccase-3 RNA | GAGTTCCTGGTTTAACTGTGAATTTAGCAATTGGCGCTTCGTTGTGAACGCC | This study |
| *E. scolopes* Laccase-3 Probe #9 | Target Laccase-3 RNA | CAGATATTTCAAACGGATAAAGAGCTCCAGCTGCGATTACTCTAAAACGATA | This study |
| *E. scolopes* Laccase-3 Probe #10 | Target Laccase-3 RNA | GTGGCTCCAATTCACAGCCGTCAGACGAGACAATCTGAAGCTTATGACCGTC | This study |
| *E. scolopes* Laccase-3 Probe #11 | Target Laccase-3 RNA | CGAATTCTGTCGTTATCCCACGAATCCAGTAGTTCGCTGGTGGTTGGTTGGC | This study |
| *E. scolopes* Laccase-3 Probe #12 | Target Laccase-3 RNA | CTTCATCAGGGGAACCTTCGTAGTGCAGAATAGCTTCAAAGGTGTGGTTTTT | This study |
| *E. scolopes* Laccase-3 Probe #13 | Target Laccase-3 RNA | TTAAGACCCCACACGGGTCATTTTCCGAGCAGTTCTTACTTGCAGAATTCGG | This study |
| *E. scolopes* Laccase-3 Probe #14 | Target Laccase-3 RNA | GCTGGTATAATGGGTTTACAGACGGCTCCTGGTATTTGCGCCCGTTTACTGA | This study |
| *E. scolopes* Laccase-3 Probe #15 | Target Laccase-3 RNA | ATATCCGATCAACTCCGCAGTCTTGCTTATCGCATAATGTGTCTACTTCGTT | This study |
| *E. scolopes* Laccase-3 Probe #16 | Target Laccase-3 RNA | CTCCTGTAGTCGTATTAAATATAGGGTATCCCATTTTTACAAGCGAAAACGA | This study |
| *E. scolopes* Laccase-3 Probe #17 | Target Laccase-3 RNA | CGGGAATGTTATCTCCACCCCAGTTGCTGTTCGACCAGGTGGCATCGTTACA | This study |
| *E. scolopes* Laccase-3 Probe #18 | Target Laccase-3 RNA | AATGCATGAACCACAGACCTGGGTTATCAGCCTTGATCCGAATAACAGCATA | This study |
| *E. scolopes* Laccase-3 Probe #19 | Target Laccase-3 RNA | GTGTGAAACTCCGACACACAGGAAAATGGGCAGGTGCCTCGGGCACATCAGC | This study |
| *E. scolopes* Laccase-3 Probe #20 | Target Laccase-3 RNA | GGGTCGTCGTGCCGTCCCTCGTTTCCATCATTGGTATGCGACTGATTGCTTC | This study |
