# Supplementary material for: The noncoding small RNA SsrA is released by Vibrio fischeri and modulates critical host responses
Source: PLoS Biol. 2020 Nov 3;18(11):e3000934. doi: 10.1371/journal.pbio.3000934 (PMC7665748; doi:10.1371/journal.pbio.3000934)
Supplement: Attachment — Submitted filename: Response to Reviewers.docx [file pbio.3000934.s017.docx]

Responses to Reviewer Comments:

**Reviewer #1**: In the manuscript entitled "The non-coding small RNA ssrA is secreted by Vibrio fischeri to modulate critical host responses," the authors show convincingly that the small RNA ssrA, a transfer messenger RNA with a well-established role in ribosome rescue, is secreted in membrane vesicles and taken up by squid light organ epithelial cells.  This attenuates the host innate immune response, allowing V. fischeri persist in the squid light organ.  This is a fascinating and important story that adds to our knowledge of the interaction of host epithelia with co0mmensals.  Some observations are underdeveloped but do not detract from the overall impact of the reported findings.  The following are specific comments:

Major comments:
1) Line 39: Reference 27 to V. cholerae OMV is not appropriate here.  This work shows that a sRNA increases V. cholerae OMV production due to a decrease in expression of the outer membrane protein OmpA.  OmpA, itself, is a virulence factor.  The increase in OmpA expression in a sRNA mutant is most likely responsible for increased pathogen virulence, and no evidence is presented that the sRNA is transferred to the host cytoplasm or that it impacts the host epithelium directly.

Reference 27 has been removed from line 39 as suggested.

2) Line 95:  Hemolymph RNAseq: The majority of bacterial RNA's found in host hemolymph were tRNA's and ribosomal RNA's.  
a) Is this a consequence of the stability of these RNA's?  Is there any evidence of RNA fragments or degradation?
A clarification has been added (line 98), indicating that we used size selection for the library preparation, focusing on the small RNA fraction. Nevertheless, based on differential coverage, we did observe degradation of the rRNA.

b) For sRNA's, did the reads include the entire sRNA or were all parts of the sRNA equally represented in the reads?  
The sRNAs had full coverage in the reads. On line 108, a clarification has been added to the text.

c) Were there any non-V. fischeri bacterial RNA's detected in the hemolymph?  In other words, are all these bacterial RNA's coming from the light organ, which is mono-colonized?

While many of these RNAs (e.g., tRNAs) are highly conserved and, thus, theoretically could be from other bacterial species, there was no evidence that any other organisms contributed them; i.e., the majority of sequences belonged exclusively to *V. fischeri* (line 96).

d) The authors state that the same RNA's were detected in OMV's regardless of what medium V. fischeri was grown in.  Is ssrA more abundantly transcribed than the other sRNA's detected in OMV's and hemolymph or is it selectively packaged into OMVs?
A comparison of cytoplasmic and OMV sRNA populations indicated no evidence of any significant selective packaging between these two compartments; i.e., all the major sRNAs were present in roughly the same proportions in both. A statement has been added to that effect (line 111). In addition, Fig S1A & A’ provide evidence that there is no differential packaging of SsrA based on growth medium.

3) Line 111: The statement here that the OMV's differ only in the absence of ssrA seems too strong given the evidence provided.  For instance, I wonder if the size and number of OMV's is the same and whether this might also affect the phenotype.

The reviewer is correct; we should have made our point more specific.. that there were no differences in the kinds or amounts of sRNA in OMVs produced by the wild type or ssrA mutant. We have added that specification to the text (line 119).

We used the total protein present in our purified OMV prep as an estimate of the amount of OMV material produced. We have not observed differences in the total amount of protein per ml of OMV preps made from cultures of the wild type or the mutant at the same OD, suggesting that the amount of OMVs produced is similar (line 124). Whether the size range of the OMVs differed was not determined.

4) Line 177: Are there other reasons in vivo light emission could be less in the ssrA mutant?  For instance, are the lux genes just as highly expressed? Is high cell density not signaled or sensed in vivo?
The cell density of symbionts in the light organ is orders of magnitude higher than (i) can be achieved in culture, and (ii) is necessary to fully induce the luminescence operon (line 208). The cited reference (53) goes through the experimental evidence and logic for concluding that oxygen limitation is likely constrained in those symbionts that aren’t producing their full potential for light emission.

5) Figure 3B: In this heat map, it looks like ssrA rescue is very incomplete.  In other words, the host response to the rescued ssrA mutant looks more like the response to the mutant itself than that to the wild-type bacterium.  Is it possible that overexpression of ssrA results in less packaging into OMV's?

It is true that, while the response of the host to the complemented strain has become more like the response to wild type, it has not fully phenocopied it. (Perhaps this is why genetic complements are included in host-response heat maps so rarely.) As is typical, the genetic complementation of the Δ*ssrA* mutant is done with a plasmid, and this one overexpresses both *ssrA* and *smpB*, which might explain why the downstream responses are not identical to those of a wild type colonization. Nevertheless, the purpose of this RNAseq experiment was to identify candidate host genes, such as laccase, whose expression could be determined with more precision in targeted qRT and HCR experiments (Fig 3-C, C’ & D). Future experiments will confirm the responses of other candidate host genes.

6) Line 281: Is it also possible that OMV's formed outside the squid are different in some way or that OMV's that lack ssrA function in a dominant negative way.  Perhaps an in vivo competition experiment would address the question of whether proximity to the epithelium is necessary for rescue by ssrA+ OMV's.
This was an excellent suggestion by two of the reviewers. Accordingly, we performed a new *in vivo* complementation experiment (added as Fig S5C) and described in the text (line 253). The results of this experiment showed that co-colonization with the wild-type strain was sufficient to complement the mutant, and restore the host-survival phenotype.

7) Line 313-Hemocyte experiments: It is not clear from this discussion whether the experiments in hemocytes are relevant to what is observed in squid light organ epithelial cells.  Shouldn't activation of an innate immune RNA sensor lead to a more robust immune response?  I'm not convinced that these experiments strengthen the story.
As stated in the text (line 334), our use of haemocytes here is analogous to using tissue culture for a targeted assay in a mammalian model; i.e., to screen for responses that are in difficult to assay locations in the body. The fact that invertebrate epithelia are non-professional phagocytes strengthens the relevance of using as a proxy phagocytic haemocytes, which have also been shown to take up OMVs. However, we recognize that the host response to SsrA might differ between haemocytes and epithelial cells and a clarification has been inserted into the main text (line 350) to emphasize that future studies will be focusing on the matter. Nevertheless, in the present study we showed that a host cell can recognize SsrA delivered naturally through symbiont OMVs; the downstream pathway(s) leading to an apparent suppression of immune response have not yet been identified.

Minor comments:
1) Line 92: "one such study…"  This wording is awkward and gives the impression that there were many similarly performed RNAseq studies of squid hemolymph, but only one gave this result.  I suspect this is not the case.

The wording on line 93 has been changed as only one RNAseq on the squid hemolymph was performed.

2) Line 145: Should this be Figure 1D rather than 1B?
The reviewer is correct; changed to Fig 1D (line 154).

3) Line 150: Should this be Figure 1E rather than 1C?
The reviewer is correct; changed to Fig 1E (line 162).

4) Line 157: This sentence is confusing.  Which signals and secreted molecules are the authors referring to? Is ssrA a signal or a bacterial effector?
The sentence has been re-worded for clarification (line 168), and to respond to Reviewer #3’s concern with the meaning of the term ‘signal’ (see below).

5) Line 170: I apologize for the nit-picking, but technically the bacterial production of MAMP's has not been measured even qualitatively.  I would say, instead, that the host response to these MAMP's is not different in the mutant.
The wording has been changed to reflect the host response, rather than the production of MAMPs (line 182).

5) Line 301:  Is the implication that the juveniles do not take in nutrients other than those in the yolk sac?  For the non-experts, it would be helpful to state this directly.
It has been clarified (line 316) that juveniles, after hatching, use their internal yolk sac as the principal source of energy while they find prey and learn to hunt.

6) Line 305:  This sentence is unclear.  What happened to a "significantly greater extent as confirmed by electron microscopy"? 
The sentence has been clarified (line 321).


**Reviewer #2**: The authors have established the squid Euprymna scolopes and its symbiont Vibrio fischeri as an outstanding model for understanding host-microbe interactions.  In particular, they have studied every stage of colonization and persistence from both the host and bacterium perspective.  This has allowed a detailed description of the genetics, biochemistry and biology of this complex interaction. In the current manuscript, they show that a small ncRNA produced by the bacteria enters host cells and manipulates the immune response toward the vibrios, thus preserving the mutualistic interaction.  Failure to produce and deliver the ncRNA results in altered innate immune signaling and reduced persistence of the bacteria, a response that is detrimental to both host and bacterium.  The authors use a variety of techniques to show that the effect of the ncRNA is on the host, not the bacterium, and to establish its localization and effects on host cells.  Overall, this study
will be of broad interest, and it provides insights into the interaction of the host with a beneficial bacterium, an area that is understudied compared to host-pathogen interactions.

Specific questions and comments :

1. SsrA is found in vesicles and there is evidence in previous studies that vesicles can deliver cargo to host cells, but is there direct evidence that SsrA enters the host cell through vesicles?  Can the RNA also be secreted into the environment and taken up by host cells, or does it require the vesicle for delivery?

As cited, we have demonstrated SsrA within OMVs, and that the host epithelium (and haemocytes) take up symbiont OMVs (Aschtgen et al 2016). However, we do not have direct evidence that all of the symbiont SsrA that enters the host cell does so within OMVs; while free-RNA might be expected to occur in the symbiont crypts as a result of possible bacterial lysis, we are not inclined to suggest that possibility since there the existing evidence is that there is no lysis (i.e., no sign of bacterial ghosts in TEMs; no evidence of dead cells; no decrease in symbiont number after growth ceases each day). Text and a new citation have been added (lines 158-161) to further strengthen that point. It is formally possible that SsrA is somehow directly secreted through the cell envelope by the bacteria and, before it is degraded, is taken up by the epithelium; however, testing that parallel hypothesis falls outside of the scope of this paper.

2.If SsrA is acting directly on host cells and is not required by the bacteria to maintain colonization, the wild type strain should be able to rescue the ssrA mutant, i.e. if the squid are infected with a 1:1 mixture of the wild type and mutant, the wild type strain should not outcompete the mutant.  I am not sure whether competition experiments can be done in this model, since the bacteria are flushed daily, but it would be interesting to see if the mutant bacteria could persist in the squid that is also exposed to the wild type.  
As described above (response to Reviewer 1, comment #6), we performed a co-colonization of the squid light organ with wild type and the Δ*ssrA* mutant, co-inoculated at a 1:1 ratio. The description of the experiment can be found at line 124, and the results in the newly added Fig S2-D. Although the wild type outcompetes the mutant strain in the light organ, the mutant persists for at least 72 h.

**Reviewer #3:** The manuscript 'The non-coding small RNA SsrA is secreted by Vibrio fischeri to modulate critical host responses' by Moriano-Gutierrez et al. studies the influence of the secreted sRNA SsrA of the squid symbiont V. fischeri on its host. The authors discover that, in absence of the ssrA gene, bacterial persistence and luminescence of the squid's light organ is much reduced. Strikingly, this phenomenon is not dependent on SmpB, the natural protein partner of the ssrA gene product, the tmRNA. Therefore, a defect in the bacterial trans-translation ribosome rescue system cannot explain the observed phenotypes. They can detect Vibrio tmRNA outside the bacteria, in host tissue, supporting their model that it is carried along with other stable transcripts into the host via outer membrane vesicles. Finally, the wild-type and the ssrA deletion strains are shown to elicit different host responses (regulated genes in the squid), with an interesting effect on genes related
to immune function. 
The manuscript is well written and presents new insight into the functions of secreted RNAs, and the reported observations are truly novel and exciting. Naturally, one wishes to see more mechanistic detail, for example, a better understanding of how tmRNA may influence host gene expression, whether bacterial tmRNA can be recovered with RIG-I, whether particular regions of the RNA are responsible for the observed phenotypes and so forth. However, we appreciate that this is not an easy experimental system, and would argue that some of those mechanistic question may be addressed in a follow-up paper. 

Major points:
*	Since the authors, at this point in time, cannot conclusively prove that SsrA is actively secreted and taken up by the host, strong statement such as "SsrA signaling" and related terms should be softened throughout the paper. True, they do show that tmRNA is present in OMVs and that it can be detected by FISH in host tissue. But this does not prove secretion, it might as well be bacterial cell lysis (which would equally explain the detection of 16S rRNA in host epithelium; Fig S3).

The reviewer is concerned with two different but related points: the use of the terms ‘signaling’ and ‘secretion’. It is true that there is an increasing number of reports of bacteria producing OMVs containing SsrA and, thus, it is unlikely that *V. fischeri* has evolved the release of SsrA-containing OMVs specifically to signal its host. Rather, it is more likely that the crypt epithelium is simply using the detection of SsrA as a cue to the presence of its symbiont, *V. fischeri*, the only bacterium that can give this cue because it is the only one that has access to the crypts. For this reason, we agree with the reviewer, and have changed references to ‘SsrA signaling’ in the text to the more host-oriented ‘SsrA sensing’.

As for proving ‘secretion’ of SsrA, it depends on whether one considers the release of cargo in OMVs as secretion, which most of the OMV community does. However, since we used ‘secretion’ only once (in the title), we have changed that to ‘release’ to avoid any possible confusion about implied mechanism.

Finally, there is no evidence for cell lysis among the symbiont population (see new text and citation at lines 158-163), which, when not growing, is in a metabolically active, growth stasis (no replication, but no decrease in numbers); in contrast, it is typical for 16S rRNA to be reported among the RNA species found in OMVs.

*	Are the effects on the host specific to the tmRNA of Vibrio fischeri? The authors should at least attempt to complement the ssrA deletion strain with ssrA genes from evolutionary related or distant bacteria, so see whether this "signaling" is a trait that evolved in this specific symbiosis.

We also find this is an interesting question and will be addressing it in future experiments, as mentioned in line 395-398. However, at this point, randomly choosing one different species or another, and either showing specificity (or not) is unlikely to provide a satisfying figure, and would only open a door we can’t properly close in this manuscript. You can be sure that a project to identify the importance of sequence, domain, length, and structure, providing insight into the mechanism, is being designed, as indicated on line 393-395.

*	Several of the figures/tables lack appropriate labeling (the labels of Fig 1C are missing the log base and it is not stated what was compared to calculate the fold change; Fig 2 contains several cut-off error bars; Fig 5B is only showing half of the error bars; Figs 2, 3C', 5B, S1A, S1A', S2B, S5C, S6A', S6B should show all data points).

We thank the reviewer for identifying these omissions. In Fig 1C, the log base has been included and the fold-change in gene-expression comparison has been added to the figure legend.

The X axis in Fig 2B, C and D has been extended as suggested; however, although the error bars seem to be cut-off in this figure, Fig 2 looks this way because it has box-and-whisker plots in all panels, and in some instances the minimum value coincides with the end of the quartile; hence, it appears as if the minimum value bar is missing.

We have added both error bars to Fig 5B. All data points are shown in the box plots whenever it did not compromise the visualization of the plot itself. Finally, we added all data points to the bar graphs in Figs 5B and S1A and S1A'.

*	Table 1 gives the number of reads for certain ncRNAs in OMVs and haemolymph. This is uninformative without knowledge of the total number of reads and should be changed to give normalized percentages instead.

As suggested, we have included the normalized percentages in Table 1.

*	Further, some of the data important for the major conclusions seem to be over/misinterpreted. In lines 113-116, the indicated difference shown in figure 2A might be statistically significant, but the decrease shown is marginal at best. Thus, the statement on line 119-120 is premature at this point. Line 174-175 and the abstract state that the ssrA knockout mutant reduces luminescence by 90% but Figure 2E does not support this statement. Similarly, all data shown in Fig 5B show strong variance but is still used for important conclusions such as that CIKS expression is only affected by tmRNA-deficient OMVs, whereas wt OMVs had no effect. Given the data presented, this seems like an over interpretation and the authors should avoid this conclusion and rephrase line 358-366 and move it to the discussion. This also means that the model shown in Fig 5C, much of which is based on Fig 5B, should be re-evaluated. Finally, the numbers of replicates for the data shown in Fig 5B are missing.

The point made in lines 121-124 is the same as that made by the reviewer.. that there are no differences in these *in vitro* phenotypes. In contrast, the 2-fold drop in abundance depicted in Fig 2A is statistically significant difference and indicates the beginning of a depletion of symbionts that is more apparent in some of the animals (note the extended lower range of points in Δ*ssrA* at 48 h); however, we agree these data are better reflected by changing ‘compromised’ to ‘affected’.

The statement in the main text (line 185) about the decreased luminescence of the *ssrA* mutant compared to WT (when in the light organ) has been modified for clarification. We apologize for that inconsistency, and thank the reviewer for noting it. The *ssrA* mutant cells, when in the light organ, only showed 20% of the WT level of luminescence.

Both Figs 5B and 5C have been modified to remove the speculative role of CIKS in the model. Similarly, there is no longer any mention of CIKS (previously, lines 363-365) in the main text, and we have remodeled that area of the Discussion (lines 381-392). Finally, as suggested, the number of biological replicates performed has been added to the legend of Fig 5B.

*	The authors exclusively focus on the function of tmRNA as a secreted molecule, given that the smpB knockout background shows similar behavior as the wt. Yet, it is important to at least discuss the possibility of tmRNA having additional functions in the bacteria that might explain the observations. Could the host effects stem from a tmRNA function as a regulatory RNA in the bacteria?

We have looked through the literature, and spoken with colleagues who work on tmRNA at the NIH, and there is apparently no published evidence to date of the tmRNA SsrA having any function in bacteria apart from its role in the ribosome-rescue system. For this reason we are reluctant to speculate that there is an unknown regulatory function for tmRNA within bacteria, and that unknown function is the reason why the mutant loses its effects on the host.

*	The Conclusions section should be changed into a proper discussion, where the authors should more critically reflect upon the strengths and weaknesses of their present model and discuss it in more detail with the available literature. On the latter, see recent work from the Cossart laboratory in Paris discovering putative RNA export and delivery of bacterial transcripts to RIG-I (PMID: 31761719, PMID: 31951583).  
We thank the reviewer for alerting us to these recent publication. The Discussion section of the presented model has been extended (lines 371 to 398) to include this and other recent literature. Interestingly, the Vogel preview supports our understanding that nothing is known about mechanisms for directly secreting bacteria RNA.

Minor points:
*	When referring to the RNA, please use tmRNA; ssrA is the gene name.

We agree that *ssrA* is the gene name and, by applying the generic convention for naming the transcript of an sRNA gene, we use SsrA (e.g., *csrB* and CsrB). While this particular sRNA is generally called ‘tmRNA’ to denote its (currently) sole function, we prefer not to use tmRNA throughout this manuscript, because it gives the false impression that, in this research, its transfer-messenger function is critical to the story. At this point, all we know is the *ssrA* transcript has a critical function that doesn’t require SmpB. However, to inform the readers of its common name, we introduce the alternate term ‘tmRNA’ in the Introduction (line 77).

*	Supplementary file 1: For easy data access, VF_XXXX locus tags might be more useful to the community than the shown GeneIDs, which do not seem to be part of the indicated annotations

Great advice. The VF_XXXX locus tags have been added to Supplementary File 1.

*	Lines 36-38: reference 16 does not seem relevant as it focuses on DNA and plasmids found in OMVs, it does not concern the host response.

That reference has been removed.

*	Lines 95-97: The authors refer to tRNAs and ncRNAs as ORFs. Please correct this. Additionally, it is odd that not a single mRNA was detected in these OMVs.

The term has been corrected from ORFs to genes. (line 96)

For our library preparation we enriched in RNAs from 85nt to 300nt. So if there are mRNAs within OMVs that are not fragmented we would not have captured them. There are only a few RNAseq studies that report mRNAs within OMVs, and they appear in very low proportions of the reads. For example: 0.44 % (Blenkiron et al. 2016) and 0.19% (Goshal et al. 2015).

*	Lines 108-110: reference 46 is only refers to OMVs and spread in mice. Please adjust statement or reference included in the indicated lines ("…as reported in other animals.").

The statement has been modified to identify mice, specifically (line 115-116).

*	Fig S2D: Why does the complementation of ssrA lead to such an increase in smpB mRNA levels?

The complement carries on a multicopy plasmid wild-type versions of both *ssrA* and *smpB*, resulting in their overexpression. An explanation of this point has been included in the legend for Fig S2E.

*	Lines 124-126: please indicate the samples per group.

As indicated, for each strain a total of 60 juvenile squid were analyzed (6 replicates of 10 squid) to compare the bacterial colonization levels of the 3 strains (wild type and its two mutant derivatives) at each of two time points (total of 360 animals in the experiment).

*	Line 113 may refer to Fig S1C.

The change in figure labeling has been made as suggested (line 119).

*	Line 145 should refer to Fig 1D (twice).

The change in figure labeling has been made as suggested (line 154).

*	Line 148: the mentioned data not shown data seem very interesting and could be included in the manuscript

The requested data have been added as a new Fig S3 (line 157).

*	Line 150 should refer to Fig 1E.

The change in figure labeling has been made as suggested (line 162)

*	Line 219: "their induction suggested that the host treats the ΔssrA colonization as an undesired infection" This statement should be re-phrased, there is currently no proof for it.

The statement has been re-phrased (line 231).

*	Line 306: "the yolk sac had significantly decreased not only in area, but also to a significantly greater extent". Please re-phrase for clarity.

This sentence has been re-worded for clarification (line 321).
