# Supplementary material for: The noncoding small RNA SsrA is released by Vibrio fischeri and modulates critical host responses
Source: PLoS Biol. 2020 Nov 3;18(11):e3000934. doi: 10.1371/journal.pbio.3000934 (PMC7665748; doi:10.1371/journal.pbio.3000934)
Supplement: Attachment — Submitted filename: Response to Reviewers.docx [file pbio.3000934.s018.docx]

**Response to Reviewers:**

Based on the positive comments on our revision (see below), we have made no changes to the manuscript.

REVIEWERS' COMMENTS:

Reviewer #1:

“The authors have addressed all the reviewer's comments in detail. This is a fascinating manuscript that, in my view, should be accepted for publication.”

Reviewer #2: [identifies herself as Shelley M. Payne]

“The revised version of the manuscript has addressed my comments and concerns.”
